# Efficacy of group training using Gross model-based emotion regulation strategies in reducing ‎suicidal ideation in patients with major depression disorder who had attempted suicide, ‎Bushehr, 1396‎ 

**Published:** 2019-08

**Authors:** Jamileh Kiani, Fatemeh Gholizadeh

**Affiliations:** ^*a*^Clinical Research development Center, Bushehr University of Medical Sciences, Bushehr, Iran.

## Abstract

**Background:**

Major depression is one of the century s most common mental illnesses, affecting 15% of the ‎population. Many factors contribute to depression including age, gender, occupation, traumatic ‎event, genetics, etc. This illness is more common in single and divorced people than married ‎ones. Due to suicidal ideation in patients, major depression is among the priorities of treatment. ‎This illness causes many problems in the families and imposes a considerable economic burden ‎on governments and families. Purpose: The present research aimed at determining the ‎effectiveness of group training using Gross model-based emotion regulation strategies in ‎reducing suicidal ideation in patients with major depression who had attempted suicide. ‎

**Methods:**

In this research, a quasi-experimental pretest posttest design with a control group was used. ‎Statistical population included patients with major depression disorder who had attempted ‎suicide and were referred to centers of psychotherapy in the city of Bushehr. From the statistical ‎population, 20 volunteers were selected and randomly assigned to two equal groups (treatment ‎and control). Following that, eight sessions of group training using Gross model-based emotion ‎regulation strategies was run on the treatment group twice a week each 90 minutes. The sessions ‎were designed based on raising conscientiousness, detecting stressful situations and promoting ‎behavioral components such as individual growth and adaptability. In this sessions, ‎communication, conceptualization, awareness and managing positive and negative emotions ‎were instructed. Individuals were supposed to learn how to comprehend the difference between ‎types of emotional functioning, communicate with others, and regulate their mood and ‎emotions. Training on emotional drain, relaxation and reverse action and finally, assessing and ‎applying learned skills in natural situations were not included in the sessions. The control group ‎did not receive any intervention.‎

**Results:**

Findings revealed that suicidal ideation was less in the treatment group than the control group ‎and the difference was statistically significant (p-value<0.001). ‎

**Conclusions:**

Difficulty in regulating emotions is one of the problems patients with major depression tackle ‎with. Training such patients how to express and manage emotions can help reducing their ‎problems. Therefore, it can be resulted that Gross model-based emotion regulation strategies is ‎effective on decreasing suicidal ideation in patients with major depression and can increase ‎their power of coping and adaptation.‎

**Keywords:**

Emotion regulation, Major depression, Suicidal ideation

